# Increased infiltration of regulatory T cells in hepatocellular carcinoma of patients with hepatitis B virus pre-S2 mutant

**DOI:** 10.1038/s41598-020-80935-5

**Published:** 2021-01-13

**Authors:** Chiao-Fang Teng, Tsai-Chung Li, Ting Wang, Da-Ching Liao, Yi-Hsuan Wen, Tzu-Hua Wu, John Wang, Han-Chieh Wu, Woei-Cherng Shyu, Ih-Jen Su, Long-Bin Jeng

**Affiliations:** 1 Graduate Institute of Biomedical Sciences, China Medical University, No. 91, Hsueh-Shih Rd., Northern Dist., Taichung City, 404 Taiwan, ROC; 2 Organ Transplantation Center, China Medical University Hospital, Taichung, Taiwan, ROC; 3 Research Center for Cancer Biology, China Medical University, No. 2, Yude Rd., Northern Dist., Taichung City, 404 Taiwan, ROC; 4 Department of Public Health, College of Public Health, China Medical University, Taichung, Taiwan, ROC; 5 Department of Healthcare Administration, College of Medical and Health Science, Asia University, Taichung, Taiwan, ROC; 6 School of Pharmacy, China Medical University, Taichung, Taiwan, ROC; 7 Department of Pathology, China Medical University Hospital, Taichung, Taiwan, ROC; 8 National Institute of Infectious Diseases and Vaccinology, National Health Research Institutes, Zhunan, Taiwan, ROC; 9 Department of Occupational Therapy, Asia University, Taichung, Taiwan, ROC; 10 Department of Neurology, China Medical University Hospital, Taichung, Taiwan, ROC; 11 Translational Medicine Research Center, China Medical University Hospital, Taichung, Taiwan, ROC; 12 Department of Biotechnology, Southern Taiwan University of Science and Technology, Tainan, Taiwan, ROC

**Keywords:** Liver cancer, Biomarkers

## Abstract

Hepatocellular carcinoma (HCC) is a frequent and deadly human cancer worldwide that is intimately associated with chronic hepatitis B virus (HBV) infection. Pre-S2 mutant is a HBV oncoprotein that plays important roles in HCC development and is linked to poor prognosis in HCC patients. However, the profiles of tumor-infiltrating lymphocytes in HCC tissues of pre-S2 mutant-positive patients remain unknown. In this study, we performed fluorescent immunohistochemistry staining to detect the infiltration of ‘anti-tumor’ cytotoxic T lymphocytes (CTLs) and ‘pro-tumor’ regulatory T cells (Tregs) in pre-S2 mutant-positive and -negative HCC patients. We showed that pre-S2 mutant-positive patients had a significantly higher infiltration of CD4^+^CD25^+^ cells and forkhead box P3 (Foxp3)-expressing cells but similar CTLs and lower granzyme B-expressing cells in HCC tissues compared with pre-S2 mutant-negative patients. Moreover, the percentage of pre-S2 plus pre-S1 + pre-S2 deletion (pre-S2 mutant) was positively correlated with the density of CD4^+^CD25^+^ cells and Foxp3-expressing cells but negatively with granzyme B-expressing cells in HCC tissues. Considering that increased intratumoral Tregs have been shown to promote tumor immune evasion, our data may provide new insights into the pathogenesis of HBV pre-S2 mutant-induced HCC and suggest that therapeutics targeting Tregs may be a promising strategy for treating pre-S2 mutant-positive high-risk patient population.

## Introduction

As one of the most common and lethal human cancers, hepatocellular carcinoma (HCC) kills approximately 700,000 people each year worldwide^1,2^. Although potentially curative treatments such as liver transplantation and surgical resection are available for HCC patients, these treatments are challenged by the scarcity of donor livers and the high rate of HCC recurrence up to 80% within 5 years after surgery, respectively, resulting in poor patient outcomes^3,4^. Moreover, the survival benefit provided by currently available nonsurgical treatments such as chemotherapeutic or molecular targeted agents is limited for HCC patients because HCC tumors exhibit high genetic heterogeneity as well as drug resistance^5,6^. Therefore, it is urgently needed to develop new therapeutics and strategies for the treatment of HCC to improve patient survival.

Chronic hepatitis B virus (HBV) infection is one of the major risk factors for HCC development, responsible for over 50% of total cases worldwide^7,8^. Our previous studies have well demonstrated that pre-S2 mutant, which harbors in-frame deletion mutations in the pre-S2 gene segment of HBV large surface protein, is an important HBV oncoprotein that can induce multiple signal pathways to promote proliferation, survival, and genomic instability of hepatocytes, eventually contributing to HCC formation in vitro and in vivo^9–12^. Chronic HBV carriers and HBV-related HCC patients, who carry pre-S2 mutant in liver tissues or blood, have been significantly associated with a higher risk of HCC development and recurrence after surgical resection, respectively^13–18^. Furthermore, we have recently developed a next-generation sequencing (NGS)-based platform for quantitative detection of pre-S deletions in plasma and identified that HCC patients with either deletion spanning pre-S2 gene segment or high percentage of pre-S2 plus pre-S1 + pre-S2 deletion, who were defined as the pre-S2 mutant-positive HCC patients, have a poorer recurrence-free survival after surgical resection^19–21^. Therefore, it is important to discover potential therapeutic strategies for treating the pre-S2 mutant-positive HCC patients.

Evasion of tumor cells from host immune surveillance in the tumor microenvironment is a critical step for the development of solid tumors, including HCC^22,23^. Tumor microenvironment is a complex and dynamic system, which comprises both cellular and subcellular components with reciprocal interplay^24^. Among the cellular components, tumor-infiltrating lymphocytes, especially the cytotoxic T lymphocytes (CTLs) and regulatory T cells (Tregs), are considered as the primary immune components, contributing to the host immune responses to solid tumors^25^. CTLs exhibit anti-tumor activities through releasing the cytotoxic granules containing perforin and granzyme B; conversely, Tregs display pro-tumor activities through impairing the capacities of CTLs, including proliferation, activation, as well as release and production of cytotoxic granule proteins^26,27^. Also, expression of the transcription factor forkhead box P3 (Foxp3) is essential for the regulatory functions of Tregs^28^. Indeed, the intratumoral balance of CTLs and Tregs has been shown to play a crucial role in the progression of HCC. Infiltration of Tregs in tumor tissues is increased and associated with defective CTLs and poor survival in HCC patients^29,30^. Moreover, several therapeutics for promoting CTLs or suppressing Tregs activities have been proposed to augment the host’s anti-tumor immunity^31,32^. Therefore, more understanding of the profiles of tumor-infiltrating lymphocytes in HCC patients has great promise in identifying optimal treatment regimes to improve their prognosis.

It has been shown that chronic HBV infection plays a key role in modulating the level and activity of tumor-infiltrating lymphocytes of the HCC tumor microenvironment^33^. Increased levels of Tregs are observed in patients with chronic HBV infection and are linked to impaired immune functions of CTLs, promoting the progression of HCC^34^. However, the profiles of CTLs and Tregs in tumor of the pre-S2 mutant-positive HCC patients remain to be clarified. In this study, we enrolled 40 HBV-related HCC patients and divided them into two groups, the pre-S2 mutant-positive and -negative groups, according to either the presence of deletion spanning pre-S2 gene segment or the percentage of pre-S2 plus pre-S1 + pre-S2 deletion. The infiltration of CTLs and Tregs in tumor tissues of these two groups of patients was detected by fluorescent immunohistochemistry (IHC) staining and was further comparatively analyzed.

## Results

### Clinicopathological profile of patients

As shown in Supplementary Table S1, the 40 HBV-related HCC patients had a median age of 54 years (range 28–78). There were 34 men and 6 women. 36 patients had available HBV DNA levels at a medium of 1.1 × 10^5^ copies/mL (range 30.1–1.5 × 10^8^) and were HBV surface antigen (HBsAg) positive. 39 patients had available tumor size at a median of 4.0 cm (range 1.5–35.0).

### Classification of patients by NGS-based pre-S genotyping

**Figure 1:**
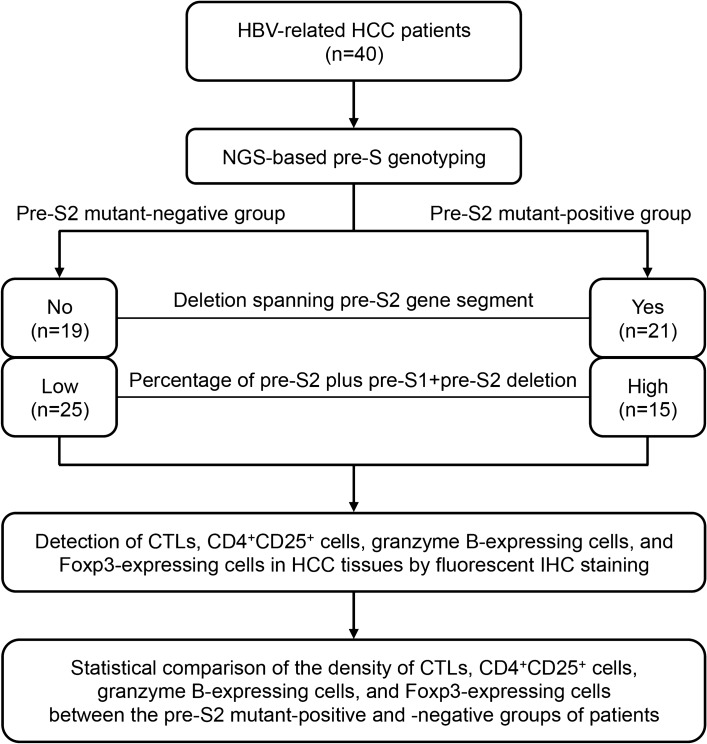
Flowchart for patient classification and quantitative analysis of CTLs, CD4^+^CD25^+^ cells, granzyme B-expressing cells, and Foxp3-expressing cells in HCC tissues in this study. By NGS-based pre-S genotyping, HBV-related HCC patients were divided into two groups, the pre-S2 mutant-positive and -negative groups, according to either the presence of deletion spanning pre-S2 gene segment or the percentage of pre-S2 plus pre-S1 + pre-S2 deletion. By fluorescent IHC staining, CTLs, CD4^+^CD25^+^ cells, granzyme B-expressing cells, and Foxp3-expressing cells in HCC tissues were detected and quantified as the density of CTLs, CD4^+^CD25^+^ cells, granzyme B-expressing cells, and Foxp3-expressing cells for comparative analysis between the two groups of patients.

In this study, patients with either deletion spanning pre-S2 gene segment or high percentage of pre-S2 plus pre-S1 + pre-S2 deletion were defined as the pre-S2 mutant-positive patients; conversely, patients without these pre-S deletions were the pre-S2 mutant-negative patients. Among the 40 HBV-related HCC patients, 21 patients were positive and 19 negative for deletion spanning pre-S2 gene segment; in addition, 15 patients had high and 25 low percentage of pre-S2 plus pre-S1 + pre-S2 deletion (Fig. 1).

### Detection and quantification of CTLs and CD4^+^CD25^+^ cells in HCC tissues of patients

**Figure 2:**
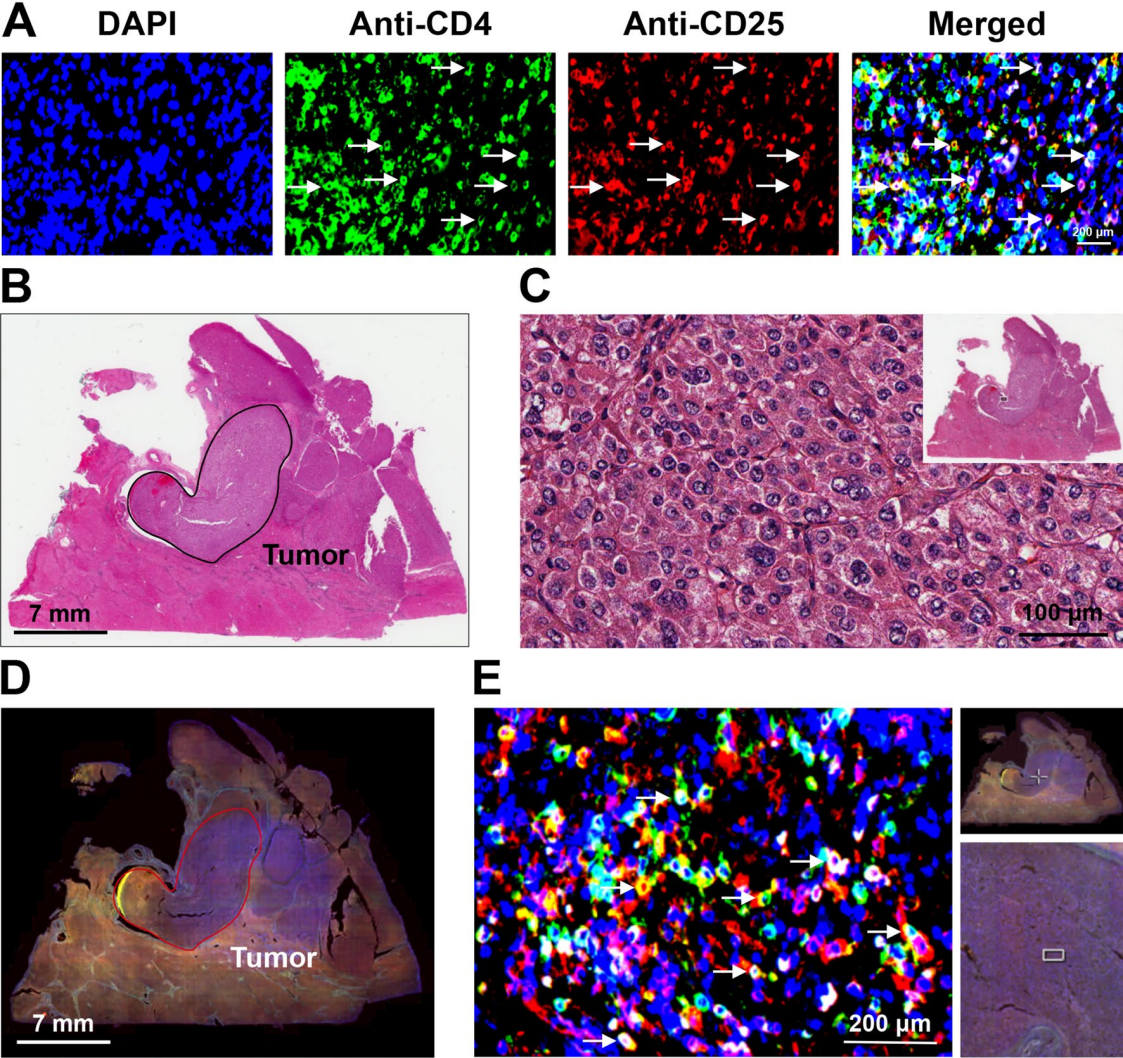
Detection and quantification of CD4^+^CD25^+^ cells in HCC tissues of patients. (**A**) CD4^+^CD25^+^ cells in liver tissues of HBV-related HCC patients were detected by fluorescent IHC staining with antibodies against CD4 (Anti-CD4) and CD25 (Anti-CD25). CD4^+^CD25^+^ cells were double positive for CD4 (green in color) and CD25 (red in color) and appeared yellow, as indicated by white arrows in the single-color and merged images. Nuclei were counterstained with DAPI (blue in color). Shown were representative results. Original magnification, ×40. Scale bar, 200 μm. (**B**) Whole-slide image of the liver tissue section stained by H&E to define the tumor region, as highlighted by the black circle. Shown was a representative image. Scale bar, 7 mm. (**C**) Magnification of the tumor region outlined by the white rectangle box in the top-right image of the H&E-stained liver tissue section. Original magnification, ×40. Scale bar, 100 μm. (**D**) Whole-slide image of the liver tissue section stained by fluorescent IHC with CD4 and CD25 antibodies. Tumor region was highlighted by the red circle. Shown was a representative merged image. Scale bar, 7 mm. (**E**) Magnification of the tumor region outlined by the white crossed lines in the top-right image and the white rectangle box in the down-right image of the fluorescent IHC-stained liver tissue section. CD4^+^CD25^+^ cells were double positive for CD4 (green in color) and CD25 (red in color) and appeared yellow, as indicated by white arrows. Nuclei were counterstained with DAPI (blue in color). Shown was a representative merged image. Original magnification, ×40. Scale bar, 200 μm.

Fluorescent IHC staining was carried out to detect CTLs and CD4^+^CD25^+^ cells in liver tissues of the 40 HBV-related HCC patients (Fig. 2A and Supplementary Figure S1A). Meanwhile, hematoxylin and eosin (H&E) staining was performed to determine the region of tumor histopathology in liver tissues (Figs. 2B,C and Supplementary Figure S1B,C). The number of CTLs and CD4^+^CD25^+^ cells in the tumor regions was then quantified as the density of CTLs and CD4^+^CD25^+^ cells in HCC tissues (Fig. 2D,E and Supplementary Figure S1D,E).

### Pre-S2 mutant-positive patients exhibited a significantly higher density of CD4^+^CD25^+^ cells but similar CTLs in HCC tissues

**Figure 3:**
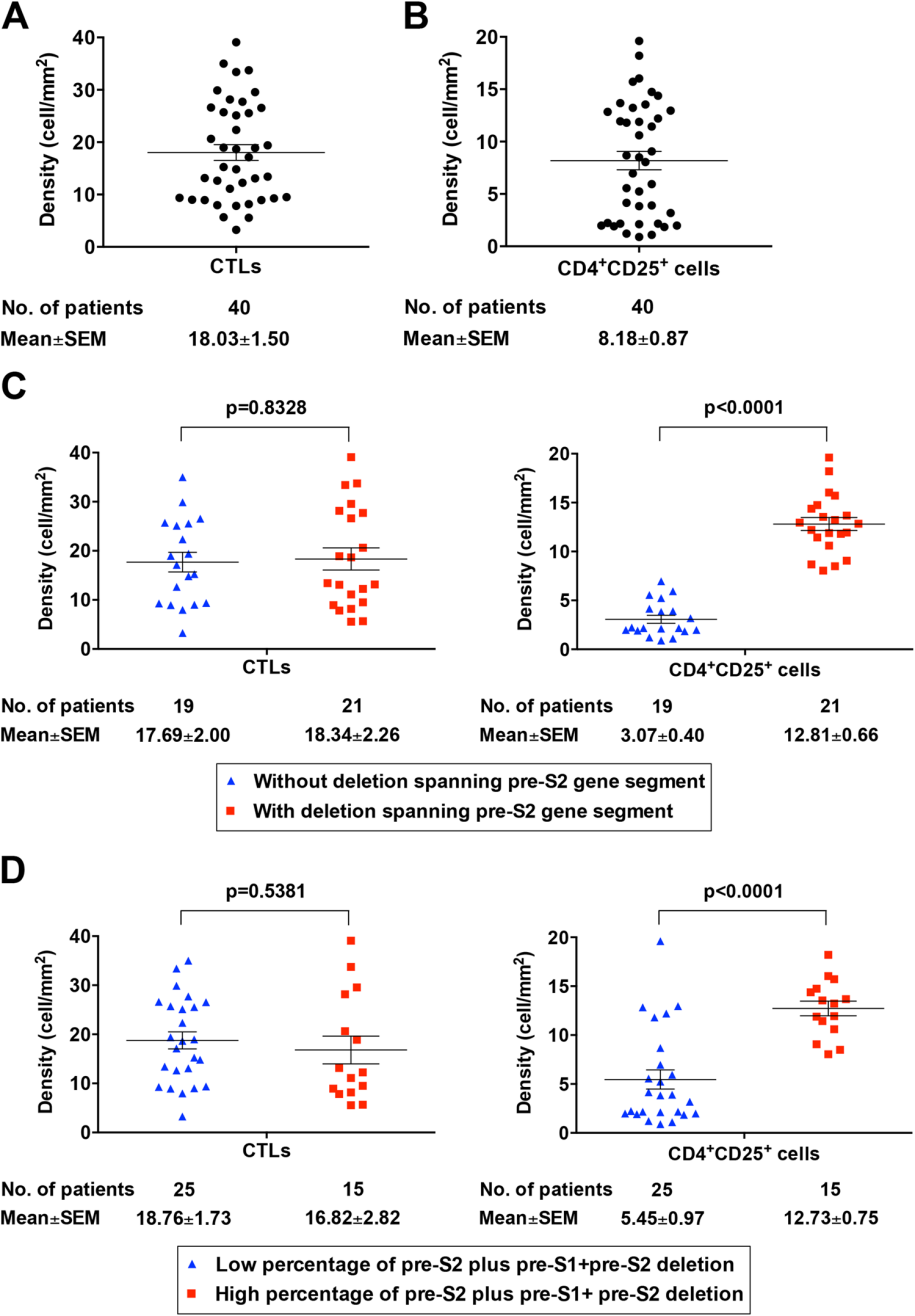
Pre-S2 mutant-positive patients exhibited a significantly higher density of CD4^+^CD25^+^ cells but similar CTLs in HCC tissues. (**A**) Density of CTLs in HCC tissues of total patients. (**B**) Density of CD4^+^CD25^+^ cells in HCC tissues of total patients. (**C**) Density of CTLs (left graph) and CD4^+^CD25^+^ cells (right graph) in HCC tissues of patients with and without deletion spanning pre-S2 gene segment. (**D**) Density of CTLs (left graph) and CD4^+^CD25^+^ cells (right graph) in HCC tissues of patients with high and low percentage of pre-S2 plus pre-S1 + pre-S2 deletion. Horizontal lines represented the mean values of the distribution. A P value < 0.05 was considered statistically significant.

The density (mean ± standard error of the mean (SEM)) of CTLs and CD4^+^CD25^+^ cells in HCC tissues of the 40 HBV-related HCC patients was 18.03 ± 1.50 and 8.18 ± 0.87 cells/mm^2^, respectively (Fig. 3A,B). Patients with either deletion spanning pre-S2 gene segment or high percentage of pre-S2 plus pre-S1 + pre-S2 deletion had a significantly higher density of CD4^+^CD25^+^ cells in HCC tissues than those without (mean ± SEM, 12.81 ± 0.66 versus 3.07 ± 0.40 cells/mm^2^, *P* < 0.0001; 12.73 ± 0.75 versus 5.45 ± 0.97 cells/mm^2^, *P* < 0.0001, respectively) (Fig. 3C,D; right graphs). However, there was no significant difference of the density of CTLs in HCC tissues between the pre-S2 mutant-positive and -negative patients (Fig. 3C,D; left graphs).

Consistently, when dividing the density of CTLs and CD4^+^CD25^+^ cells in HCC tissues into the high and low density by using the mean density as a cut-off value, patients with either deletion spanning pre-S2 gene segment or high percentage of pre-S2 plus pre-S1 + pre-S2 deletion were significantly associated with high density of Tregs in HCC tissues (*P* < 0.0001) (Tables 1 and 2). No significant correlation was observed between the density of CTLs in HCC tissues and either the presence of deletion spanning pre-S2 gene segment or the high percentage of pre-S2 plus pre-S1 + pre-S2 deletion in patients (Tables 1 and 2). The correlation between the pre-S deletions and other clinicopathological factors was summarized in Supplementary Tables S2 and S3.

**Table 1:** Clinicopathological correlation of the deletion spanning pre-S2 gene segment in 40 HBV-related HCC patients.

Characteristics^a^	Negative (no. of patients (%))	Positive (no. of patients (%))	P value^b^
**Density of CTLs**^**c**^	19 (100)	21 (100)	
High	9 (47)	10 (48)	0.2482
Low	10 (53)	11 (52)	
**Density of CD4**^**+**^**CD25**^**+**^** cells**^**d**^	19 (100)	21 (100)	
High	0 (0)	20 (95)	< 0.0001***
Low	19 (100)	1 (5)	
**Density of granzyme B-expressing cells**^**e**^	19 (100)	21 (100)	
High	16 (84)	3 (14)	< 0.0001***
Low	3 (16)	18 (86)	
**Density of Foxp3-expressing cells**^**f**^	19 (100)	21 (100)	
High	0 (0)	15 (71)	< 0.0001***
Low	19 (100)	6 (29)	

*HBV* hepatitis B virus, *HCC* hepatocellular carcinoma, *CTLs* cytotoxic T lymphocytes, *Foxp3* forkhead box P3, *HBeAg* hepatitis B e antigen, *AST* aspartate aminotransferase, *ALT* alanine aminotransferase, *AFP* alpha-fetoprotein, *CLIP* Cancer of the Liver Italian Program, *BCLC* Barcelona Clinic Liver Cancer, *AJCC* American Joint Committee on Cancer, *TNM* tumor-node-metastasis.

***P value < 0.001.

^a^Only patients with available data were analyzed.

^b^P value was determined by the chi-square test.

^c^The density of CTLs in tumor tissues above the mean density (18.03) was defined as high density.

^d^The density of CD4^+^CD25^+^ cells in tumor tissues above the mean density (8.18) was defined as high density.

^e^The density of granzyme B-expressing cells in tumor tissues above the mean density (5.53) was defined as high density.

^f^The density of Foxp3-expressing cells in tumor tissues above the mean density (35.58) was defined as high density.

**Table 2:** Clinicopathological correlation of the percentage of pre-S2 plus pre-S1 + pre-S2 deletion in 40 HBV-related HCC patients.

Characteristics^a^	Low (no. of patients (%))	High (no. of patients (%))^b^	P value^c^
**Density of CTLs**^**d**^	25 (100)	15 (100)	
High	13 (52)	6 (40)	0.1983
Low	12 (48)	9 (60)	
**Density of CD4**^**+**^**CD25**^**+**^** cells**^**e**^	25 (100)	15 (100)	
High	6 (24)	14 (93)	< 0.0001***
Low	19 (76)	1 (7)	
**Density of granzyme B-expressing cells**^**f**^	25 (100)	15 (100)	
High	17 (68)	2 (13)	0.0009***
Low	8 (32)	13 (83)	
**Density of Foxp3-expressing cells**^**g**^	25 (100)	15 (100)	
High	3 (12)	12 (80)	< 0.0001***
Low	22 (88)	3 (20)	

*HBV* hepatitis B virus, *HCC* hepatocellular carcinoma, *CTLs* cytotoxic T lymphocytes, *Foxp3* forkhead box P3, *HBeAg* hepatitis B e antigen, *AST* aspartate aminotransferase, *ALT* alanine aminotransferase, *AFP* alpha-fetoprotein, *CLIP* Cancer of the Liver Italian Program, *BCLC* Barcelona Clinic Liver Cancer, *AJCC* American Joint Committee on Cancer, *TNM* tumor-node-metastasis.

***P value < 0.001.

^a^Only patients with available data were analyzed.

^b^The percentage of pre-S2 plus pre-S1 + pre-S2 deletion above 24.995 was defined as high percentage.

^c^P value was determined by the chi-square test.

^d^The density of CTLs in tumor tissues above the mean density (18.03) was defined as high density.

^e^The density of CD4^+^CD25^+^ cells in tumor tissues above the mean density (8.18) was defined as high density.

^f^The density of granzyme B-expressing cells in tumor tissues above the mean density (5.53) was defined as high density.

^g^The density of Foxp3-expressing cells in tumor tissues above the mean density (35.58) was defined as high density.

### Pre-S2 mutant-positive patients displayed a significantly lower density of granzyme B- but higher density of Foxp3-expressing cells in HCC tissues

**Figure 4:**
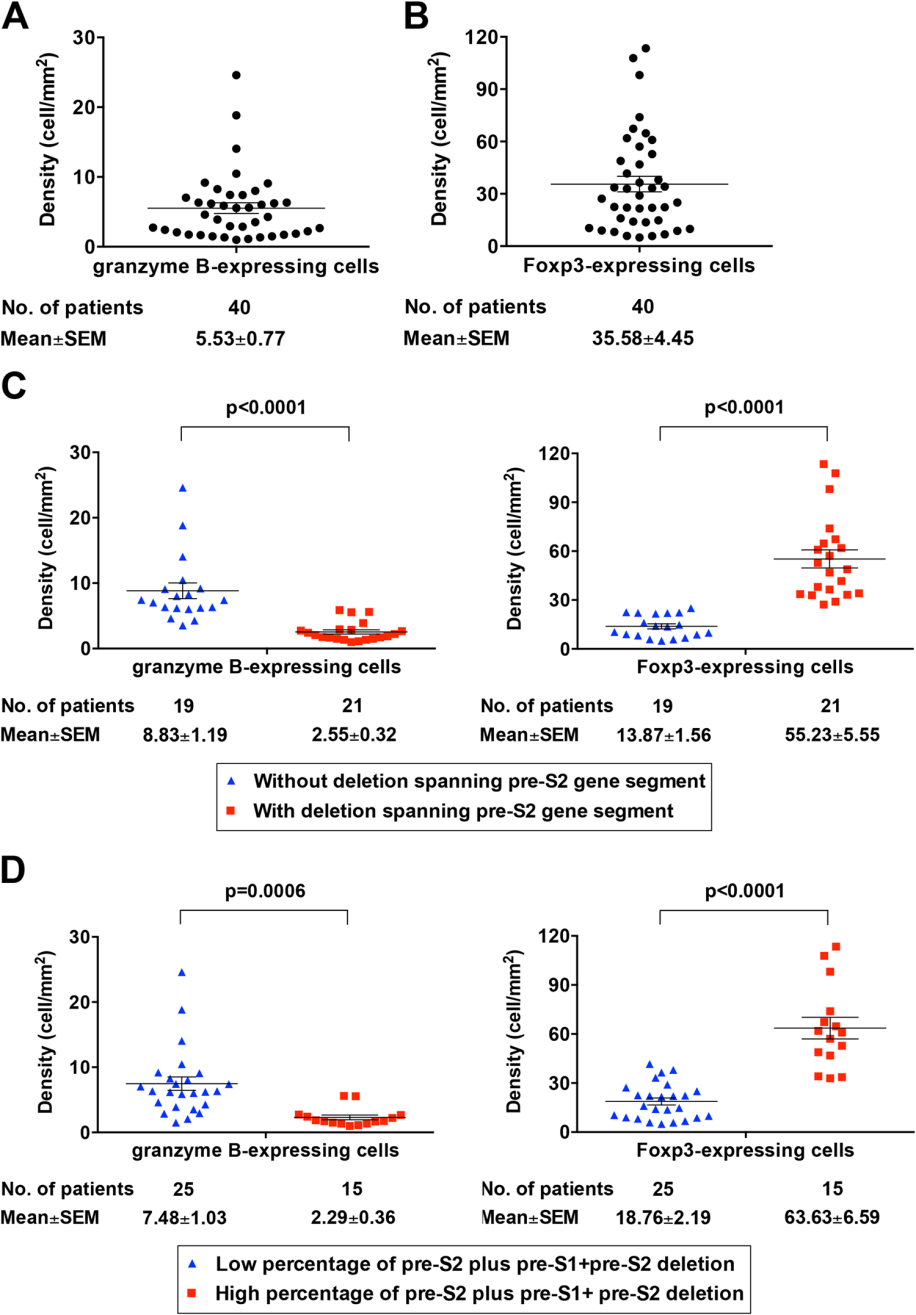
Pre-S2 mutant-positive patients displayed a significantly lower density of granzyme B- but higher density of Foxp3-expressing cells in HCC tissues. (**A**) Density of granzyme B-expressing cells in HCC tissues of total patients. (**B**) Density of Foxp3-expressing cells in HCC tissues of total patients. (**C**) Density of granzyme B-expressing cells (left graph) and Foxp3-expressing cells (right graph) in HCC tissues of patients with and without deletion spanning pre-S2 gene segment. (**D**) Density of granzyme B-expressing cells (left graph) and Foxp3-expressing cells (right graph) in HCC tissues of patients with high and low percentage of pre-S2 plus pre-S1 + pre-S2 deletion. Horizontal lines represented the mean values of the distribution. A P value < 0.05 was considered statistically significant.

Considering that granzyme B and Foxp3 are essential for CTLs- and Tregs-mediated immune responses, respectively^26,28^, their expression in HCC tissues was examined as shown in Supplementary Figures S2 and S3. As shown in Fig. 4A,B, the density (mean ± SEM) of granzyme B- and Foxp3-expressing cells in HCC tissues of the 40 HBV-related HCC patients was 5.53 ± 0.77 and 35.58 ± 4.45 cells/mm^2^, respectively. The density of granzyme B-expressing cells in HCC tissues was significantly lower in patients with either deletion spanning pre-S2 gene segment or high percentage of pre-S2 plus pre-S1 + pre-S2 deletion than those without (mean ± SEM, 2.55 ± 0.32 versus 8.83 ± 1.19 cells/mm^2^, *P* < 0.0001; 2.29 ± 0.36 versus 7.48 ± 1.03 cells/mm^2^, *P* = 0.0006, respectively) (Fig. 4C,D; left graphs). Conversely, the density of Foxp3-expressing cells in HCC tissues was significantly higher in patients with either deletion spanning pre-S2 gene segment or high percentage of pre-S2 plus pre-S1 + pre-S2 deletion than those without (mean ± SEM, 55.23 ± 5.55 versus 13.87 ± 1.56 cells/mm^2^, *P* < 0.0001; 63.63 ± 6.59 versus 18.76 ± 2.19 cells/mm^2^, *P* < 0.0001, respectively) (Fig. 4C,D; right graphs).

The same correlation was observed when the density of granzyme B- and Foxp3-expressing cells in HCC tissues was divided into the high and low density based on the mean density. As shown in Tables 1 and 2, patients with either deletion spanning pre-S2 gene segment or high percentage of pre-S2 plus pre-S1 + pre-S2 deletion were significantly associated with low density of granzyme B- and high density of Foxp3-expressing cells in HCC tissues (*P* < 0.0001).

### Percentage of pre-S2 plus pre-S1 + pre-S2 deletion was positively correlated with the density of CD4^+^CD25^+^ cells and Foxp3-expressing cells but negatively with granzyme B-expressing cells in HCC tissues

**Figure 5:**
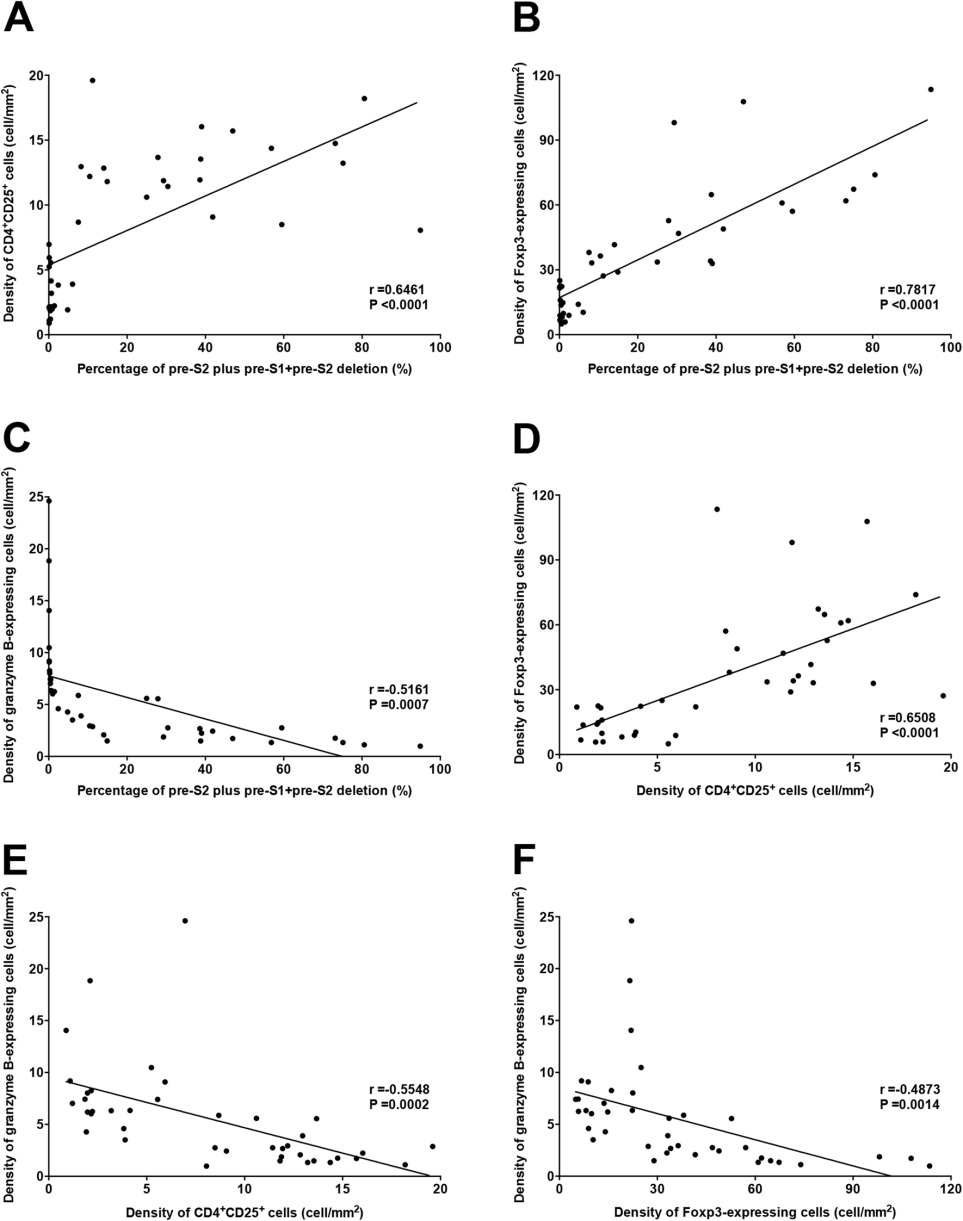
Percentage of pre-S2 plus pre-S1 + pre-S2 deletion was positively correlated with the density of CD4^+^CD25^+^ cells and Foxp3-expressing cells but negatively with granzyme B-expressing cells in HCC tissues. (**A**) Correlation between the percentage of pre-S2 plus pre-S1 + pre-S2 deletion and the density of CD4^+^CD25^+^ cells in HCC tissues. (**B**) Correlation between the percentage of pre-S2 plus pre-S1 + pre-S2 deletion and the density of Foxp3-expressing cells in HCC tissues. (**C**) Correlation between the percentage of pre-S2 plus pre-S1 + pre-S2 deletion and the density of granzyme B-expressing cells in HCC tissues. (**D**) Correlation between the density of CD4^+^CD25^+^ cells and Foxp3-expressing cells in HCC tissues. (**E**) Correlation between the density of CD4^+^CD25^+^ cells and granzyme B-expressing cells in HCC tissues. (**F**) Correlation between the density of Foxp3- and granzyme B-expressing cells in HCC tissues. The associated linear regression line was depicted in 40 HBV-related HCC patients in each graph. A P value < 0.05 was considered statistically significant.

The relationship between the percentage of pre-S2 plus pre-S1 + pre-S2 deletion and the density of CD4^+^CD25^+^ cells, Foxp3-expressing cells, and granzyme B-expressing cells was assessed. As shown in Fig. 5A,B, there was a high positive correlation between the percentage of pre-S2 plus pre-S1 + pre-S2 deletion and the density of CD4^+^CD25^+^ cells and Foxp3-expressing cells in HCC tissues (Pearson’s correlation coefficient (r) = 0.6461; *P* < 0.0001; r = 0.7817, *P* < 0.0001, respectively). Conversely, there was a high negative correlation between the percentage of pre-S2 plus pre-S1 + pre-S2 deletion and the density of granzyme B-expressing cells in HCC tissues (r = -0.5161; *P* = 0.0007) (Fig. 5C). Furthermore, the density of CD4^+^CD25^+^ cells showed a high positive correlation with the density of Foxp3-expressing cells but a high negative correlation with the density of granzyme B-expressing cells in HCC tissues (r = 0.6508, *P* < 0.0001; r = − 0.5548, *P* = 0.0002, respectively) (Fig. 5D,E). A high negative correlation was observed between the density of Foxp3- and granzyme B-expressing cells in HCC tissues (r = -0.4873, *P* = 0.0014) (Fig. 5F).

## Discussion

Despite considerable progress in the treatment of HCC, the prognosis of HBV-related HCC patients remains poor, highlighting an urgent need for new therapeutics and strategies to improve patient survival^35,36^. Previously, we have identified that the HBV-related HCC patients with either deletion spanning pre-S2 gene segment or high percentage of pre-S2 plus pre-S1 + pre-S2 deletion (the pre-S2 mutant-positive HCC patients) have a worse prognosis than those without (the pre-S2 mutant-negative HCC patients)^21^. In this study, we further demonstrated that the pre-S2 mutant-positive patients exhibited a higher density of CD4^+^CD25^+^ cells and Foxp3-expressing cells concurrent with a similar CTLs but lower granzyme B-expressing cells in HCC tissues compared with the pre-S2 mutant-negative patients. Increased Tregs and decreased CTLs in number and activity in HCC tissues have been shown to promote tumor immune evasion and closely associated with poor patient outcomes^29^. Our results therefore suggest that enhancing CTLs or suppressing Tregs activities in HCC tumor microenvironment may be a promising strategy for treating and improving prognosis of the pre-S2 mutant-positive high-risk patient population.

Transforming growth factor-β1 (TGF-β1) is a crucial cytokine mediating immune suppression in the tumor microenvironment^37^. Several mechanisms have been proposed to explain the immune regulatory functions of TGF-β1. First, TGF-β1 is requisite to direct the differentiation of naive CD4^+^ T cells into Tregs^38^. Second, TGF-β1 is necessary to promote the activities of Tregs through upregulating Foxp3 expression^39^. Next, TGF-β1 is efficient to induce the recruitment of Tregs to tumor sites^40^. Moreover, TGF-β1 is important to suppress the activities of CTLs through inhibiting the expression of cytotoxic genes, such as granzyme B^41^. Indeed, HCC tumor cells-secreted TGF-β1 plays a critical role in tumor progression by suppressing anti-tumor immunity in the tumor microenvironment through the aforementioned mechanisms^42^. Furthermore, our previous study has revealed that overexpression of pre-S2 mutant in the human hepatoma HuH-7 cell line displays increased secretion of TGF-β1 in the culture supernatant^43^. These findings, together with the results of this study, therefore suggest that the pre-S2 mutant-enhanced TGF-β1 secretion from HCC tumor cells may be probably one of the possible explanations for the increased infiltration and activity of Tregs as well as decreased activity of CTLs in HCC tissues of the pre-S2 mutant-positive patients. Additionally, considering that increased ratio of intratumoral Tregs to CTLs in number and activity has been closely linked to disease progression and poor prognosis of HCC patients with chronic HBV infection^29,30^, the results of this study may not only provide a new insight into the pre-S2 mutant-induced tumorigenesis, during which pre-S2 mutant may facilitate survival and propagation of tumor cells through shaping the tumor immune microenvironment from tumor eradication (low Tregs and high CTLs) into tumor evasion (high Tregs and low CTLs), but also offer a possible explanation for the higher risk of HCC development and recurrence in the pre-S2 mutant-positive patients. Further investigation of the underlying mechanisms is needed and promising to identify novel therapeutic targets for the pre-S2 mutant-positive HCC patients.

Tregs have been shown to hamper anti-tumor immune responses in the tumor microenvironment through various mechanisms^44^. First, Tregs constitutively express CD25 to form the high-affinity interleukin (IL)-2 receptor to capture exogenous IL-2 cytokine for their survival, thereby limiting the amount of IL-2 in the surroundings available for activation and proliferation of CTLs^45^. Second, Tregs express high levels of the cytotoxic T lymphocyte antigen 4 (CTLA-4) molecules to down-regulate the expression of co-stimulatory molecules CD80 and CD86 in antigen-presenting cells (APCs), thus inhibiting their capacity to activate CTLs^46^. Moreover, Tregs produce and secrete immune-suppressive cytokines, such as TGF-β and IL-10, to down-modulate the functions of APCs and CTLs^47^. Studies have shown that the number of Tregs along with levels of TGF-β and IL-10 is significantly higher in HCC patients and associated with a worse prognosis^29,30,48^. Furthermore, selective depletion or blockade of Tregs by monoclonal antibodies targeting the aforementioned Tregs-expressed surface molecules or cytokines has emerged as a potential therapeutic strategy to augment anti-tumor immunity against HCC^31,32^. Considering that the pre-S2 mutant-positive HCC patients exhibit high intratumoral Tregs infiltration and activity and have a poor prognosis after surgical resection, the therapeutics targeting Tregs may hold promise as an alternative treatment option for this patient population. Furthermore, the results of this study showed that higher percentage of pre-S2 plus pre-S1 + pre-S2 deletion was correlated with a higher density of CD4^+^CD25^+^ cells and Foxp3-expressing cells in HCC tissues. Because higher levels of pre-S2 mutant have been associated with poorer prognosis in HCC patients^16,18,21^, the results of this study may probably not only provide a possible explanation for the poorer prognosis but also highlight a higher need for the Tregs therapy in patients with higher levels of pre-S2 mutant.

There are some limitations to this study. One limitation concerns the surface markers used to detect Tregs in HCC tissues. In this study, Tregs were defined as the cells double positive for CD4 and CD25 (CD4^+^CD25^+^ cells) and the expression of Foxp3 alone was additionally detected for assessing the activity of Tregs. However, it has been shown that CD4 and CD25 are also expressed in nonregulatory effector T cells and Foxp3 is also transiently expressed in activated nonregulatory effector T cells^49^. Indeed, the results of this study revealed that the mean density of Foxp3-expressing cells was higher than the mean density of CD4^+^CD25^+^ cells in HCC tissues of pre-S2 mutant-positive patients, suggesting that Foxp3 expression does not exclusively occur in CD4^+^CD25^+^ cells. Also, reports have shown evidence for Foxp3 expression in the cells positive for CD4 but negative for CD25 (CD4^+^CD25^−^ cells)^50,51^. Therefore, detection of the cells concurrently expressing CD4, CD25, and Foxp3 will allow for more specific analysis of the infiltration of Tregs in HCC tissues of pre-S2 mutant-positive patients. Another limitation is the lack of analysis of the extrinsic regulatory molecules for Tregs. For example, the programmed death 1 (PD-1)/programmed death ligand 1 (PD-L1) axis has been shown to play a crucial role in induction of the development and function of Tregs through enhancing Foxp3 expression^52^. More comprehensive profiling of the regulators of Tregs will better dissect the role of Tregs in regulating the tumor immune microenvironment of pre-S2 mutant-positive patients. Furthermore, it is important and worthwhile to clarify the regulation of Tregs by pre-S2 mutant, elucidate the underlying mechanisms, and validate the potential of therapeutics targeting Tregs in treatment of pre-S2 mutant-induced tumorigenesis in cell and animal studies. In addition, although the clinicopathological characteristics of the cohort of 40 HCC patients analyzed in this study coincide with the representative features of a large population of HCC patients in Taiwan^53^, a large cohort of patients from different clinical centers are needed to further validate the finding of this study in clinical practice. Even so, this study is the first study to our knowledge to provide insights into the profiles of tumor-infiltrating lymphocytes in HCC tissues of pre-S2 mutant-positive patients. Moreover, the NGS-based quantitative detection of pre-S2 mutant makes it possible to evaluate the correlation between the level of pre-S2 mutant and the density of intratumoral Tregs in HCC patients.

In conclusion, in this study, we showed an increased infiltration of CD4^+^CD25^+^ cells and Foxp3-expressing cells in HCC tissues of the patients with HBV pre-S2 mutant. The results may provide new insights into the pathogenesis and therapeutics of HBV-related HCC, especially the pre-S2 mutant-positive HCC.

## Methods

### Patient specimens

The plasma and formalin-fixed and paraffin-embedded (FFPE) liver tissues from 40 HBV-related HCC patients were retrospectively collected at China Medical University Hospital (Taichung, Taiwan), from Jan 2006 to Jul 2017, under the approval of the China Medical University & Hospital Research Ethics Committee. The clinicopathological data of the patients were also retrieved. All research was performed in accordance with relevant guidelines and regulations and the informed consent was obtained from all participants.

### Detection of pre-S2 mutant in plasma

Pre-S2 mutant in plasma of HCC patients was detected by the NGS-based method we established previously^19^. Briefly, the pre-S gene was amplified from plasma DNA by polymerase chain reaction, followed by NGS analysis (Illumina, San Diego, CA, USA). The percentage of wild-type and mutant forms of pre-S gene was determined with a customized script. According to the pre-S genotyping result, patients could be divided into two groups, the pre-S2 mutant-positive and -negative groups, in terms of either the presence of deletion spanning pre-S2 gene segment or the percentage of pre-S2 plus pre-S1 + pre-S2 deletion (Supplementary Table S4). The presence of deletion spanning pre-S2 gene segment was defined as the percentage of either one of the two mutant forms of pre-S gene, the pre-S2 deletion and the pre-S1 + pre-S2 deletion, above a cut-off of 5.049%. The high percentage of pre-S2 plus pre-S1 + pre-S2 deletion was defined as the total percentage of pre-S2 deletion and pre-S1 + pre-S2 deletion above a cut-off of 24.995%. These criteria for patient classification were used because the patients identified with these cut-off percentages, who were collectively defined as the pre-S2 mutant-positive patients, have been shown to have a poorer recurrence-free survival after surgical resection in our previous report^21^. Conversely, the patients without the presence of deletion spanning pre-S2 gene segment or with low percentage of pre-S2 plus pre-S1 + pre-S2 deletion were collectively defined as the pre-S2 mutant-negative patients.

### Detection of CTLs and Tregs in liver tissues

CTLs and Tregs in liver tissues of HCC patients were detected by fluorescent IHC staining as previously described^54^. Briefly, FFPE liver tissues were cut into 4-μm-thick sections. The tissue sections were stained with the primary antibodies anti-CD3 (ab16669; Abcam, Cambridge, UK) together with anti-CD8 (MA5-13473; Invitrogen, Carlsbad, CA, USA) for CTLs or anti-CD4 (ab133616; Abcam) together with anti-CD25 (PA0305; Leica Biosystems, Newcastle, UK) for Tregs, followed by the secondary antibodies Alexa Fluor 488-conjugated goat anti-rabbit IgG (A11008; Invitrogen) together with Alexa Fluor 555-conjugated goat anti-mouse IgG (A-21424; Invitrogen). For staining granzyme B and Foxp3, the tissue sections were incubated with the primary antibodies anti-granzyme B (ab4059; Abcam) and anti-Foxp3 (ab4059; Abcam), followed by the secondary antibodies Alexa Fluor 488-conjugated goat anti-rabbit IgG (A11008; Invitrogen) and Alexa Fluor 555-conjugated goat anti-mouse IgG (A-21424; Invitrogen), respectively. DAPI (4′,6-diamidino-2-phenylindole; Invitrogen) was used to counterstain the nuclei. Furthermore, the tissue sections were stained with H&E to examine histopathology to define the tumor regions. Whole-slide images of the fluorescent IHC- and H&E-stained slides were captured with the 3DHistech Pannoramic SCAN II scanner (3DHistech, Budapest, Hungary) and Aperio CS2 scanner (Leica Biosystems, Buffalo Grove, IL, USA), respectively. 10 independent microscopic fields (original magnification, ×40) with the most abundant CTLs or CD4^+^CD25^+^ cells as well as granzyme B- or Foxp3-expressing cells in the tumor region of each patient’s tissue section were selected. The total number of CTLs or CD4^+^CD25^+^ cells as well as granzyme B- or Foxp3-expressing cells in the ten selected microscopic fields (area, 1.566 mm^2^ per field) of each patient’s tissue section was counted and further calculated as the density (number of cells per mm^2^) for statistical analysis.

### Statistical analysis

The unpaired *t*-test was used to analyze the difference of the density of CTLs, CD4^+^CD25^+^ cells, granzyme B-expressing cells, and Foxp3-expressing cells in tumor tissues between the pre-S2 mutant-positive and -negative groups of patients; data were expressed as the mean with the SEM error bar. The chi-square test was applied to assess the correlation of the density of CTLs, CD4^+^CD25^+^ cells, granzyme B-expressing cells, and Foxp3-expressing cells in tumor tissues with clinicopathological data between the pre-S2 mutant-positive and -negative groups of patients. The relationship between the density of CTLs, CD4^+^CD25^+^ cells, granzyme B-expressing cells, and Foxp3-expressing cells in tumor tissues and the percentage of pre-S2 plus pre-S1 + pre-S2 deletion was determined by calculating the Pearson’s correlation coefficient (r), where r > 0.9 denotes very high positive correlation; r > 0.7, high positive; r > 0.50, moderate positive; r > 0.30, low positive; and r > 0, negligible correlation^55^. In the case of − 1 < r < 0, it indicates a negative correlation corresponding to the different extents. A P value < 0.05 was considered statistically significant.

## Supplementary Information

Supplementary Legends.

Supplementary Figure S1.

Supplementary Figure S2.

Supplementary Figure S3.

Supplementary Tables.

## Data Availability

All data generated or analyzed during this study are included in this published article and its “Supplementary Information Files”.
