# Supplementary material for: Increased infiltration of regulatory T cells in hepatocellular carcinoma of patients with hepatitis B virus pre-S2 mutant
Source: Sci Rep. 2021 Jan 13;11:1136. doi: 10.1038/s41598-020-80935-5 (PMC7807072; doi:10.1038/s41598-020-80935-5)
Supplement: Supplementary file 1 — Supplementary Legends. [file 41598_2020_80935_MOESM1_ESM.pdf]

Increased Infiltration of Regulatory T Cells in Hepatocellular Carcinoma of Patients with Hepatitis B Virus Pre-S2 Mutant

Chiao-Fang Teng,[1,2,3*] Tsai-Chung Li,[4,5] Ting Wang,[2] Da-Ching Liao,[6] Yi-Hsuan Wen,[6] Tzu-Hua Wu,[2] John Wang,[7] Han-Chieh Wu,[8] Woei-Cherng Shyu,[1,9,10,11] Ih-Jen Su,[12] and Long-Bin Jeng[2*]

[1]Graduate Institute of Biomedical Sciences, China Medical University, Taichung, Taiwan
[2]Organ Transplantation Center, China Medical University Hospital, Taichung, Taiwan
[3]Research Center for Cancer Biology, China Medical University, Taichung, Taiwan
[4]Department of Public Health, College of Public Health, China Medical University, Taichung, Taiwan
[5]Department of Healthcare Administration, College of Medical and Health Science, Asia University, Taichung, Taiwan
[6]School of Pharmacy, China Medical University, Taichung, Taiwan
[7]Department of Pathology, China Medical University Hospital, Taichung, Taiwan
[8]National Institute of Infectious Diseases and Vaccinology, National Health Research Institutes, Zhunan, Taiwan
[9]Department of Occupational Therapy, Asia University, Taichung, Taiwan
[10]Department of Neurology, China Medical University Hospital, Taichung, Taiwan
[11]Translational Medicine Research Center, China Medical University Hospital, Taichung, Taiwan
[12]Department of Biotechnology, Southern Taiwan University of Science and Technology, Tainan, Taiwan
[*]Chiao-Fang Teng and Long-Bin Jeng are corresponding authors

Supplementary Figure Legends

Supplementary Figure S1. Detection and quantification of CTLs in HCC tissues of patients. (A) CTLs in liver tissues of HBV-related HCC patients were detected by fluorescent IHC staining with antibodies against CD3 (Anti-CD3) and CD8 (Anti-CD8). CTLs that were double positive for CD3 (green in color) and CD8 (red in color) appeared yellow, as indicated by white arrows in the single-color and merged images. Nuclei were counterstained with DAPI (blue in color). Shown were representative results. Original magnification, ×40. Scale bar, 200 μm. (B) Whole-slide image of the liver tissue section stained by H&E to define the tumor region, as highlighted by the black circle. Shown was a representative image. Scale bar, 5 mm. (C) Magnification of the tumor region outlined by the white rectangle box in the top-right image of the H&E-stained liver tissue section. Original magnification, ×40. Scale bar, 100 μm. (D) Whole-slide image of the liver tissue section stained by fluorescent IHC with CD3 and CD8 antibodies. Tumor region was highlighted by the red circle. Shown was a representative merged image. Scale bar, 5 mm. (E) Magnification of the tumor region outlined by the white crossed lines in the top-right image and the white rectangle box in the down-right image of the fluorescent

IHC-stained liver tissue section. CTLs were double positive for CD3 (green in color) and CD8 (red in color) and appeared yellow, as indicated by white arrows. Nuclei were counterstained with DAPI (blue in color). Shown was a representative merged image. Original magnification, ×40. Scale bar, 200 μm.

Supplementary Figure S2. Detection and quantification of granzyme B-expressing cells in HCC tissues of patients. (A) Granzyme B in liver tissues of HBV-related HCC patients was detected by fluorescent IHC staining with granzyme B antibody (Anti-granzyme B). Cells that were positive for granzyme B (green in color) were indicated by white arrows in the single-color and merged images. Nuclei were counterstained with DAPI (blue in color). Shown were representative results. Original magnification, ×40. Scale bar, 200 μm. (B) Whole-slide image of the liver tissue section stained by H&E to define the tumor region, as highlighted by the black circle. Shown was a representative image. Scale bar, 5 mm. (C) Magnification of the tumor region outlined by the white rectangle box in the top-right image of the H&E-stained liver tissue section. Original magnification, ×40. Scale bar, 100 μm. (D) Whole-slide image of the liver tissue section stained by fluorescent IHC with

granzyme B antibody. Tumor region was highlighted by the red circle. Shown was a representative merged image. Scale bar, 5 mm. (E) Magnification of the tumor region outlined by the white rectangle box in the right image of the fluorescent IHC-stained liver tissue section. Granzyme B-expressing cells appeared green as indicated by white arrows. Nuclei were counterstained with DAPI (blue in color). Shown was a representative merged image. Original magnification, ×40. Scale bar, 200 μm.

Supplementary Figure S3. Detection and quantification of Foxp3-expressing cells in HCC tissues of patients. (A) Foxp3 in liver tissues of HBV-related HCC patients was detected by fluorescent IHC staining with Foxp3 antibody (Anti-Foxp3). Cells that were positive for Foxp3 (green in color) were indicated by white arrows in the single-color and merged images. Counterstaining of Foxp3-positive nuclei (green in color) with DAPI (blue in color) appeared cyan. Shown were representative results. Original magnification, ×40. Scale bar, 200 μm. (B) Whole-slide image of the liver tissue section stained by H&E to define the tumor region, as highlighted by the black circle. Shown was a representative image. Scale bar, 6 mm. (C) Magnification of the tumor region outlined by the white rectangle box in the top-right image of the

H&E-stained liver tissue section. Original magnification, ×40. Scale bar, 100 μm. (D) Whole-slide image of the liver tissue section stained by fluorescent IHC with Foxp3 antibody. Tumor region was highlighted by the red circle. Shown was a representative merged image. Scale bar, 5 mm. (E) Magnification of the tumor region outlined by the white rectangle box in the right image of the fluorescent IHC-stained liver tissue section. Foxp3 (green in color)-expressing cells were counterstained with DAPI (blue in color) and appeared cyan in nuclei as indicated by white arrows. Shown was a representative merged image. Original magnification, ×40. Scale bar, 200 μm.
