# Supplementary material for: Increased infiltration of regulatory T cells in hepatocellular carcinoma of patients with hepatitis B virus pre-S2 mutant
Source: Sci Rep. 2021 Jan 13;11:1136. doi: 10.1038/s41598-020-80935-5 (PMC7807072; doi:10.1038/s41598-020-80935-5)

Increased Infiltration of Regulatory T Cells in Hepatocellular Carcinoma of Patients with Hepatitis B Virus Pre-S2 Mutant

Chiao-Fang Teng,[1,2,3*] Tsai-Chung Li,[4,5] Ting Wang,[2] Da-Ching Liao,[6] Yi-Hsuan Wen,[6] Tzu-Hua Wu,[2] John Wang,[7] Han-Chieh Wu,[8] Woei-Cherng Shyu,[1,9,10,11] Ih-Jen Su,[12] and Long-Bin Jeng[2*]

[1]Graduate Institute of Biomedical Sciences, China Medical University, Taichung, Taiwan
[2]Organ Transplantation Center, China Medical University Hospital, Taichung, Taiwan
[3]Research Center for Cancer Biology, China Medical University, Taichung, Taiwan
[4]Department of Public Health, College of Public Health, China Medical University, Taichung, Taiwan
[5]Department of Healthcare Administration, College of Medical and Health Science, Asia University, Taichung, Taiwan
[6]School of Pharmacy, China Medical University, Taichung, Taiwan
[7]Department of Pathology, China Medical University Hospital, Taichung, Taiwan
[8]National Institute of Infectious Diseases and Vaccinology, National Health Research Institutes, Zhunan, Taiwan
[9]Department of Occupational Therapy, Asia University, Taichung, Taiwan
[10]Department of Neurology, China Medical University Hospital, Taichung, Taiwan
[11]Translational Medicine Research Center, China Medical University Hospital, Taichung, Taiwan
[12]Department of Biotechnology, Southern Taiwan University of Science and Technology, Tainan, Taiwan
[*]Chiao-Fang Teng and Long-Bin Jeng are corresponding authors

Supplementary Figure S1

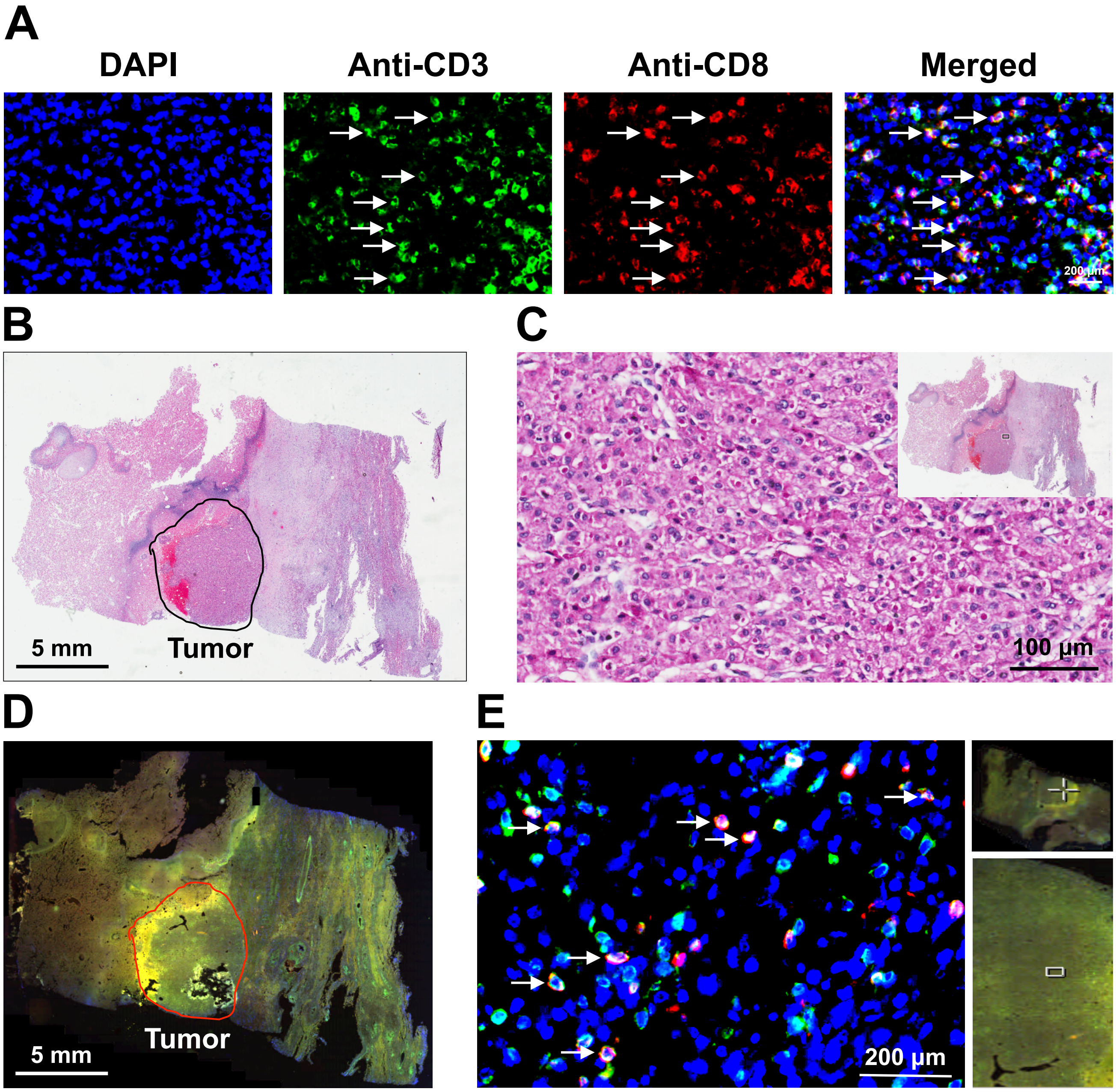

Supplement: Supplementary file 2 — Supplementary Figure S1. [file 41598_2020_80935_MOESM2_ESM.pdf]
