# Supplementary material for: Increased infiltration of regulatory T cells in hepatocellular carcinoma of patients with hepatitis B virus pre-S2 mutant
Source: Sci Rep. 2021 Jan 13;11:1136. doi: 10.1038/s41598-020-80935-5 (PMC7807072; doi:10.1038/s41598-020-80935-5)
Supplement: Supplementary file 5 — Supplementary Tables. [file 41598_2020_80935_MOESM5_ESM.docx]

**Supplementary Table S1. Clinicopathological characteristics of 40 HBV-related HCC patients enrolled in this study**

| Characteristics^a^ | No. of Patients | Median (Range) |
| --- | --- | --- |
| Age (years)  >50  ≤50 | 40  29  11 | 54 (28-78)  58 (51-78)  47 (28-49) |
| Gender (men/women) | 34/6 |  |
| Smoking (yes/no) | 14/16 |  |
| Alcohol (yes/no) | 9/31 |  |
| HBsAg (positive/negative/NA) | 36/0/4 |  |
| HBeAg (positive/negative) | 6/34 |  |
| HBV genotype (B/C) | 31/9 |  |
| HBV DNA (copies/mL) (20-1.47×10^8^/<20/NA)^b^  >1×10^4^  ≤1×10^4^ | 36/2/2  24  12 | 1.1×10^5^ (30.1-1.5×10^8^)^c^  4.5×10^5^ (1.2×10^4^-1.5×10^8^)  4.2×10^2^ (30.1-6.3×10^3^) |
| Albumin (g/dL)  >3.8  ≤3.8 | 40  18  22 | 3.8 (2.0-4.9)  4.2 (3.9-4.9)  3.4 (2.0-3.8) |
| AST (U/L)  >34  ≤34 | 40  32  8 | 37.5 (3-305)  64.5 (35-290)  25.0 (14-34) |
| ALT (U/L)  >40  ≤40 | 40  26  14 | 55.5 (13-292)  80.0 (42-292)  33.0 (13-40) |
| AFP (ng/mL) (≤54000/>54000)^d^  >400  ≤400 | 35/5  10  25 | 37.7 (1.4-4550.0)^e^  823.6 (412.3-4550.0)  20.2 (1.4-280.7) |
| Tumor size (cm)  >5  ≤5 | 39  14  25 | 4.0 (1.5-35.0)  10.0 (5.5-35.0)  3.0 (1.5-11.0) |
| Tumor encapsulation (yes/no/NA) | 30/9/1 |  |
| Satellite nodule (yes/no) | 7/33 |  |
| Lymph node involvement (yes/no) | 6/34 |  |
| Portal vein thrombosis (yes/no) | 1/39 |  |
| Vascular invasion (yes/no) | 18/22 |  |
| Distant metastasis (yes/no) | 4/36 |  |
| Steatosis grade (0/1/2/3/NA) | 23/9/1/0/7 |  |
| Metavir inflammation score (0/1/2/3/NA) | 5/29/1/0/5 |  |
| Ishak fibrosis score (0/1/2/3/4/5/6/NA) | 1/6/8/9/10/1/4/1 |  |
| Child-Pugh cirrhosis score (A/B/C) | 30/7/3 |  |
| CLIP score (0/1/2/3/4/5/6) | 16/14/6/2/1/0/1 |  |
| BCLC stage (A/B/C/D) | 25/7/5/3 |  |
| AJCC TNM stage (I/II/IIIA/IIIB/IIIC/IVA/IVB) | 17/14/4/1/3/0/1 |  |

^a^Only patients with available data were analyzed.

^b^HBV DNA was measured with a detection range of 20 to 1.47×10^8^ copies/mL.

^c,e^Only data within the detection range were analyzed.

^d^AFP was measured with the highest detection limit of 54000 ng/mL.

Abbreviations: HBV, hepatitis B virus; HCC, hepatocellular carcinoma; HBeAg, hepatitis B e antigen; NA, not available; AST, aspartate aminotransferase; ALT, alanine aminotransferase; AFP, alpha-fetoprotein; CLIP, Cancer of the Liver Italian Program; BCLC, Barcelona Clinic Liver Cancer; AJCC, American Joint Committee on Cancer; TNM, tumor-node-metastasis.

**Supplementary Table S2. Clinicopathological correlation of the deletion spanning pre-S2 gene segment in 40 HBV-related HCC patients**

| Characteristics^a^ | Negative (No. of Patients (%)) | Positive (No. of Patients (%)) | P value^b^ |
| --- | --- | --- | --- |
| Age (years)  >50  ≤50 | 19 (100)  16 (84)  3 (16) | 21 (100)  13 (62)  8 (38) | 0.0853 |
| Gender  men  women | 19 (100)  16 (84)  3 (16) | 21 (100)  18 (86)  3 (14) | 0.3358 |
| Smoking  yes  no | 19 (100)  7 (37)  12 (63) | 21 (100)  7 (33)  14 (67) | 0.2525 |
| Alcohol  yes  no | 19 (100)  2 (11)  17 (89) | 21 (100)  7 (33)  14 (67) | 0.0727 |
| HBsAg^c^  positive  negative | 17 (100)  17 (100)  0 (0) | 19 (100)  19 (100)  0 (0) |  |
| HBeAg  positive  negative | 19 (100)  2 (11)  17 (89) | 21 (100)  4 (19)  17 (81) | 0.2666 |
| HBV genotype  B  C | 19 (100)  15 (79)  4 (21) | 21 (100)  16 (76)  5 (24) | 0.2884 |
| HBV DNA (copies/mL)  >1×10^4^  ≤1×10^4^ | 18 (100)  12 (67)  6 (33) | 18 (100)  12 (67)  6 (33) | 0.2753 |
| Albumin (g/dL)  >3.8  ≤3.8 | 19 (100)  11 (58)  8 (42) | 21 (100)  7 (33)  14 (67) | 0.0775 |
| AST (U/L)  >34  ≤34 | 19 (100)  17 (89)  2 (11) | 21 (100)  15 (71)  6 (29) | 0.1207 |
| ALT (U/L)  >40  ≤40 | 19 (100)  13 (68)  6 (32) | 21 (100)  13 (62)  8 (38) | 0.2379 |
| AFP (ng/mL)  >400  ≤400 | 19 (100)  5 (26)  14 (74) | 21 (100)  10 (48)  11 (52) | 0.1020 |
| Tumor size (cm)  >5  ≤5 | 19 (100)  7 (37)  12 (63) | 21 (100)  7 (33)  14 (67) | 0.2525 |
| Tumor encapsulation  yes  no | 19 (100)  16 (84)  3 (16) | 20 (100)  14 (70)  6 (30) | 0.1772 |
| Satellite nodule  yes  no | 19 (100)  2 (11)  17 (89) | 21 (100)  5 (24)  16 (76) | 0.1866 |
| Lymph node involvement  yes  no | 19 (100)  4 (21)  15 (79) | 21 (100)  2 (10)  19 (90) | 0.2121 |
| Portal vein thrombosis  yes  no | 19 (100)  1 (5)  18 (95) | 21 (100)  0 (0)  21 (100) | 0.4750 |
| Vascular invasion  yes  no | 19 (100)  8 (42)  11 (58) | 21 (100)  10 (48)  11 (52) | 0.2351 |
| Distant metastasis  yes  no | 19 (100)  1 (5)  18 (95) | 21 (100)  3 (14)  18 (86) | 0.2765 |
| Steatosis grade  2/3  0/1 | 19 (100)  1 (5)  18 (95) | 14 (100)  0 (0)  14 (100) | 0.5758 |
| Metavir inflammation score  2/3  0/1 | 18 (100)  0 (0)  18 (100) | 17 (100)  1 (6)  16 (94) | 0.4857 |
| Ishak fibrosis score  4/5/6  0/1/2/3 | 19 (100)  7 (37)  12 (63) | 20 (100)  8 (40)  12 (60) | 0.2525 |
| Child-Pugh cirrhosis score  B/C  A | 19 (100)  4 (21)  15 (79) | 21 (100)  6 (29)  15 (71) | 0.2481 |
| CLIP score  4/5/6  0/1/2/3 | 19 (100)  2 (11)  17 (89) | 21 (100)  0 (0)  21 (100) | 0.2192 |
| BCLC stage  C/D  A/B | 19 (100)  3 (16)  16 (84) | 21 (100)  5 (24)  16 (76) | 0.2666 |
| AJCC TNM stage  IIIA/IIIB/IIIC/IVA/IVB  I/II | 19 (100)  4 (21)  15 (79) | 21 (100)  5 (24)  16 (76) | 0.2884 |

^a^Only patients with available data were analyzed.

^b^P value was determined by the chi-square test.

^c^There were no patients negative for HBsAg for analysis.

***, P value<0.001.

Abbreviations: HBV, hepatitis B virus; HCC, hepatocellular carcinoma; HBeAg, hepatitis B e antigen; AST, aspartate aminotransferase; ALT, alanine aminotransferase; AFP, alpha-fetoprotein; CLIP, Cancer of the Liver Italian Program; BCLC, Barcelona Clinic Liver Cancer; AJCC, American Joint Committee on Cancer; TNM, tumor-node-metastasis.

**Supplementary Table S3. Clinicopathological correlation of the percentage of pre-S2 plus pre-S1+pre-S2 deletion in 40 HBV-related HCC patients**

| Characteristics^a^ | Low (No. of Patients (%)) | High (No. of Patients (%))^b^ | P value^c^ |
| --- | --- | --- | --- |
| Age (years)  >50  ≤50 | 25 (100)  22 (88)  3 (12) | 15 (100)  7 (47)  8 (53) | 0.0064** |
| Gender  men  women | 25 (100)  21 (84)  4 (16) | 15 (100)  13 (87)  2 (13) | 0.3460 |
| Smoking  yes  no | 25 (100)  10 (40)  15 (60) | 15 (100)  4 (27)  11 (73) | 0.1923 |
| Alcohol  yes  no | 25 (100)  5 (20)  20 (80) | 15 (100)  4 (27)  11 (73) | 0.2652 |
| HBsAg^d^  positive  negative | 22 (100)  22 (100)  0 (0) | 14 (100)  14 (100)  0 (0) |  |
| HBeAg  positive  negative | 25 (100)  3 (12)  22 (88) | 15 (100)  3 (20)  12 (80) | 0.2726 |
| HBV genotype  B  C | 25 (100)  19 (76)  6 (24) | 15 (100)  12 (80)  3 (20) | 0.2947 |
| HBV DNA (copies/mL)  >1×10^4^  ≤1×10^4^ | 24 (100)  16 (46)  8 (54) | 12 (100)  8 (50)  4 (50) | 0.2909 |
| Albumin (g/dL)  >3.8  ≤3.8 | 25 (100)  12 (48)  13 (52) | 15 (100)  6 (40)  9 (60) | 0.2296 |
| AST (U/L)  >34  ≤34 | 25 (100)  21 (84)  4 (16) | 15 (100)  11 (73)  4 (27) | 0.2245 |
| ALT (U/L)  >40  ≤40 | 25 (100)  15 (60)  10 (40) | 15 (100)  11 (73)  4 (27) | 0.1923 |
| AFP (ng/mL)  >400  ≤400 | 25 (100)  8 (32)  17 (68) | 15 (100)  7 (47)  8 (53) | 0.1730 |
| Tumor size (cm)  >5  ≤5 | 25 (100)  9 (36)  16 (64) | 15 (100)  5 (33)  10 (67) | 0.2644 |
| Tumor encapsulation  yes  no | 25 (100)  21 (84)  4 (16) | 15 (100)  9 (60)  5 (40) | 0.1195 |
| Satellite nodule  yes  no | 25 (100)  2 (8)  23 (92) | 15 (100)  5 (33)  10 (67) | 0.0483* |
| Lymph node involvement  yes  no | 25 (100)  6 (24)  19 (76) | 15 (100)  0 (0)  15 (100) | 0.0461* |
| Portal vein thrombosis  yes  no | 25 (100)  1 (4)  24 (96) | 15 (100)  0 (0)  15 (100) | 0.6250 |
| Vascular invasion  yes  no | 25 (100)  9 (36)  16 (64) | 15 (100)  9 (60)  6 (40) | 0.0902 |
| Distant metastasis  yes  no | 25 (100)  1 (4)  24 (96) | 15 (100)  3 (20)  12 (80) | 0.1245 |
| Steatosis grade  2/3  0/1 | 23 (100)  1 (4)  22 (96) | 10 (100)  0 (0)  10 (100) | 0.6970 |
| Metavir inflammation score  2/3  0/1 | 23 (100)  0 (0)  23 (100) | 12 (100)  1 (8)  11 (92) | 0.3429 |
| Ishak fibrosis score  4/5/6  0/1/2/3 | 25 (100)  8 (32)  17 (68) | 14 (100)  7 (50)  7 (50) | 0.1476 |
| Child-Pugh cirrhosis score  B/C  A | 25 (100)  4 (16)  21 (84) | 15 (100)  6 (40)  9 (60) | 0.0747 |
| CLIP score  4/5/6  0/1/2/3 | 25 (100)  2 (8)  23 (92) | 15 (100)  0 (0)  15 (100) | 0.3846 |
| BCLC stage  C/D  A/B | 25 (100)  4 (16)  21 (84) | 15 (100)  4 (27)  11 (73) | 0.2245 |
| AJCC TNM stage  IIIA/IIIB/IIIC/IVA/IVB  I/II | 25 (100)  4 (16)  21 (84) | 15 (100)  5 (33)  10 (67) | 0.1389 |

^a^Only patients with available data were analyzed.

^b^The percentage of pre-S2 plus pre-S1+pre-S2 deletion above 24.995 was defined as high percentage.

^c^P value was determined by the chi-square test.

^d^There were no patients negative for HBsAg for analysis.

*, P value<0.05; **, P value<0.01; ***, P value<0.001.

Abbreviations: HBV, hepatitis B virus; HCC, hepatocellular carcinoma; HBeAg, hepatitis B e antigen; AST, aspartate aminotransferase; ALT, alanine aminotransferase; AFP, alpha-fetoprotein; CLIP, Cancer of the Liver Italian Program; BCLC, Barcelona Clinic Liver Cancer; AJCC, American Joint Committee on Cancer; TNM, tumor-node-metastasis.

**Supplementary Table S4.** **List of the pre-S genotyping result by NGS-based analysis in 40 HBV-related HCC patients**

| Patient No. | Pre-S deletion type (%)^a^ | Deletion spanning pre-S2 gene segment | Percentage of pre-S2 plus pre-S1+pre-S2 deletion^c^ |
| --- | --- | --- | --- |
| 1 | 1. **wild-type (99.278)^b^**  2. pre-S1 del (0.660)  3. pre-S2 del (0.053)  4. pre-S1+pre-S2 del (0.009) | negative | low |
| 2 | 1. **wild-type (96.592)**  2. pre-S1 del (2.906)  3. pre-S2 del (0.469)  4. pre-S1+pre-S2 del (0.033) | negative | low |
| 3 | 1. **wild-type (99.000)**  2. pre-S1 del (0.927)  3. pre-S2 del (0.062)  4. pre-S1+pre-S2 del (0.012) | negative | low |
| 4 | 1. **wild-type (97.851)**  2. pre-S1 del (2.037)  3. pre-S2 del (0.108)  4. pre-S1+pre-S2 del (0.005) | negative | low |
| 5 | 1. **wild-type (98.964)**  2. pre-S1 del (0.943)  3. pre-S2 del (0.084)  4. pre-S1+pre-S2 del (0.009) | negative | low |

**Supplementary Table S4. List of the pre-S genotyping result by NGS-based analysis in 40 HBV-related HCC patients (continued)**

| Patient No. | Pre-S deletion type (%)^a^ | Deletion spanning pre-S2 gene segment | Percentage of pre-S2 plus pre-S1+pre-S2 deletion^c^ |
| --- | --- | --- | --- |
| 6 | 1. **wild-type (97.781)**  2. pre-S1 del (2.099)  3. pre-S2 del (0.112)  4. pre-S1+pre-S2 del (0.008) | negative | low |
| 7 | 1. **wild-type (96.830)**  2. pre-S1 del (1.728)  3. pre-S2 del (1.341)  4. pre-S1+pre-S2 del (0.102) | negative | low |
| 8 | 1. **wild-type (98.571)**  2. pre-S1 del (1.173)  3. pre-S2 del (0.178)  4. pre-S1+pre-S2 del (0.078) | negative | low |
| 9 | 1. **wild-type (96.915)**  2. pre-S1 del (2.701)  3. pre-S2 del (0.364)  4. pre-S1+pre-S2 del (0.020) | negative | low |
| 10 | 1. **wild-type (98.069)**  2. pre-S1 del (1.263)  3. pre-S2 del (0.609)  4. pre-S1+pre-S2 del (0.059) | negative | low |

**Supplementary Table S4. List of the pre-S genotyping result by NGS-based analysis in 40 HBV-related HCC patients (continued)**

| Patient No. | Pre-S deletion type (%)^a^ | Deletion spanning pre-S2 gene segment | Percentage of pre-S2 plus pre-S1+pre-S2 deletion^c^ |
| --- | --- | --- | --- |
| 11 | 1. **pre-S1 del (92.118)**  2. **wild-type (7.278)**  3. pre-S2 del (0.372)  4. pre-S1+pre-S2 del (0.231) | negative | low |
| 12 | 1. **pre-S1 del (75.241)**  2. **wild-type (22.338)**  3. pre-S2 del (1.891)  4. pre-S1+pre-S2 del (0.530) | negative | low |
| 13 | 1. **wild-type (71.590)**  2. **pre-S1 del (28.270)**  3. pre-S2 del (0.129)  4. pre-S1+pre-S2 del (0.011) | negative | low |
| 14 | 1. **pre-S1 del (75.109)**  2. **wild-type (23.879)**  3. pre-S1+pre-S2 del (0.687)  4. pre-S2 del (0.325) | negative | low |
| 15 | 1. **pre-S1 del (86.404)**  2. **wild-type (12.695)**  3. pre-S2 del (0.737)  4. pre-S1+pre-S2 del (0.163) | negative | low |

**Supplementary Table S4. List of the pre-S genotyping result by NGS-based analysis in 40 HBV-related HCC patients (continued)**

| Patient No. | Pre-S deletion type (%)^a^ | Deletion spanning pre-S2 gene segment | Percentage of pre-S2 plus pre-S1+pre-S2 deletion^c^ |
| --- | --- | --- | --- |
| 16 | 1. **wild-type (82.957)**  2. **pre-S1 del (10.988)**  3. pre-S2 del (4.178)  4. pre-S1+pre-S2 del (1.877) | negative | low |
| 17 | 1. **wild-type (93.746)**  2. **pre-S1 del (5.846)**  3. pre-S2 del (0.398)  4. pre-S1+pre-S2 del (0.018) | negative | low |
| 18 | 1. **pre-S1 del (76.151)**  2. **wild-type (19.034)**  3. pre-S2 del (4.624)  4. pre-S1+pre-S2 del (0.192) | negative | low |
| 19 | 1. **wild-type (94.701)**  2. **pre-S1 del (5.086)**  3. pre-S2 del (0.137)  4. pre-S1+pre-S2 del (0.077) | negative | low |
| 20 | 1. **wild-type (58.461)**  2. **pre-S2 del (37.194)**  3. pre-S1 del (2.939)  4. pre-S1+pre-S2 del (1.407) | positive | high |

**Supplementary Table S4. List of the pre-S genotyping result by NGS-based analysis in 40 HBV-related HCC patients (continued)**

| Patient No. | Pre-S deletion type (%)^a^ | Deletion spanning pre-S2 gene segment | Percentage of pre-S2 plus pre-S1+pre-S2 deletion^c^ |
| --- | --- | --- | --- |
| 21 | 1. **wild-type (59.263)**  2. **pre-S2 del (37.934)**  3. pre-S1 del (1.940)  4. pre-S1+pre-S2 del (0.862) | positive | high |
| 22 | 1. **pre-S2 del (56.155)**  2. **wild-type (42.610)**  3. pre-S1+pre-S2 del (0.718)  4. pre-S1 del (0.516) | positive | high |
| 23 | 1. **pre-S2 del (94.816)**  2. **wild-type (5.049)**  3. pre-S1+pre-S2 del (0.070)  4. pre-S1 del (0.066) | positive | high |
| 24 | 1. **wild-type (65.077)**  2. **pre-S2 del (28.468)**  3. pre-S1 del (4.516)  4. pre-S1+pre-S2 del (1.940) | positive | high |
| 25 | 1. **wild-type (91.356)**  2. **pre-S2 del (8.015)**  3. pre-S1 del (0.379)  4. pre-S1+pre-S2 del (0.251) | positive | low |

**Supplementary Table S4. List of the pre-S genotyping result by NGS-based analysis in 40 HBV-related HCC patients (continued)**

| Patient No. | Pre-S deletion type (%)^a^ | Deletion spanning pre-S2 gene segment | Percentage of pre-S2 plus pre-S1+pre-S2 deletion^c^ |
| --- | --- | --- | --- |
| 26 | 1. **wild-type (46.914)**  2. **pre-S1 del (45.517)**  3. **pre-S2 del (6.834)**  4. pre-S1+pre-S2 del (0.735) | positive | low |
| 27 | 1. **pre-S1 del (76.228)**  2. **wild-type (12.583)**  3. **pre-S2 del (10.622)**  4. pre-S1+pre-S2 del (0.567) | positive | low |
| 28 | 1. **pre-S1+pre-S2 del (46.237)**  2. **pre-S2 del (26.927)**  3. **pre-S1 del (14.368)**  4. **wild-type (12.467)** | positive | high |
| 29 | 1. **wild-type (69.001)**  2. **pre-S1 del (20.530)**  3. **pre-S2 del (9.463)**  4. pre-S1+pre-S2 del (1.006) | positive | low |
| 30 | 1. **wild-type (50.938)**  2. **pre-S1 del (19.760)**  3. **pre-S1+pre-S2 del (15.021)**  4. **pre-S2 del (14.280)** | positive | high |

**Supplementary Table S4. List of the pre-S genotyping result by NGS-based analysis in 40 HBV-related HCC patients (continued)**

| Patient No. | Pre-S deletion type (%)^a^ | Deletion spanning pre-S2 gene segment | Percentage of pre-S2 plus pre-S1+pre-S2 deletion^c^ |
| --- | --- | --- | --- |
| 31 | 1. **wild-type (75.230)**  2. **pre-S1+pre-S2 del (13.878)**  3. **pre-S1 del (10.730)**  4. pre-S2 del (0.162) | positive | low |
| 32 | 1. **pre-S2 del (41.477)**  2. **pre-S1+pre-S2 del (39.126)**  3. **wild-type (12.348)**  4. **pre-S1 del (7.048)** | positive | high |
| 33 | 1. **pre-S1+pre-S2 del** **(45.703)**  2. **pre-S2 del (29.458)**  3. **wild-type (14.926)**  4. **pre-S1 del (9.913)** | positive | high |
| 34 | 1. **wild-type (30.944)**  2. **pre-S2 del (30.409)**  3. **pre-S1+pre-S2 del (29.105)**  4. **pre-S1 (9.542)** | positive | high |
| 35 | 1. **pre-S1 del (40.086)**  2. **wild-type (34.919)**  3. **pre-S2 del (16.065)**  4. **pre-S1+pre-S2 del (8.930)** | positive | high |

**Supplementary Table S4. List of the pre-S genotyping result by NGS-based analysis in 40 HBV-related HCC patients (continued)**

| Patient No. | Pre-S deletion type (%)^a^ | Deletion spanning pre-S2 gene segment | Percentage of pre-S2 plus pre-S1+pre-S2 deletion^c^ |
| --- | --- | --- | --- |
| 36 | 1. **pre-S1 del (29.181)**  2. **wild-type (23.836)**  3. **pre-S2 del (23.645)**  4. **pre-S1+pre-S2 del (23.338)** | positive | high |
| 37 | 1. **wild-type (30.973)**  2. **pre-S1+pre-S2 del (27.774)**  3. **pre-S1 del (27.161)**  4. **pre-S2 del (14.091)** | positive | high |
| 38 | 1. **pre-S1 del (43.130)**  2. **wild-type (41.965)**  3. **pre-S2 del (9.508)**  4. **pre-S1+pre-S2 del (5.397)** | positive | low |
| 39 | 1. **wild-type (36.868)**  2. **pre-S1 del (35.238)**  3. **pre-S2 del (21.130)**  4. **pre-S1+pre-S2 del (6.763)** | positive | high |
| 40 | 1. **wild-type (58.984)**  2. **pre-S2 del (34.533)**  3. pre-S1+pre-S2 del (4.497)  4. pre-S1 (1.986) | positive | high |

**Supplementary Table S4. List of the pre-S genotyping result by NGS-based analysis in 40 HBV-related HCC patients (continued)**

^a^The total percentage of pre-S gene DNA in each type of pre-S deletion was shown in descending order.

^b^The pre-S deletion type above the cut-off percentage (5.049) was shown in bold.

^c^The percentage of pre-S2 plus pre-S1+pre-S2 deletion above 24.995 was defined as high percentage.

Abbreviations: NGS, next-generation sequencing; HBV, hepatitis B virus; HCC, hepatocellular carcinoma; del, deletion.
